# The Impact of Artificial Sweeteners on Body Weight Control and Glucose Homeostasis

**DOI:** 10.3389/fnut.2020.598340

**Published:** 2021-01-07

**Authors:** Michelle D. Pang, Gijs H. Goossens, Ellen E. Blaak

**Affiliations:** Department of Human Biology, NUTRIM School of Nutrition and Translational Research in Metabolism, Maastricht University Medical Center+, Maastricht, Netherlands

**Keywords:** artificial sweeteners, obesity, type 2 diabetes mellitus, insulin resistance, gut microbiota

## Abstract

A poor diet is one of the leading causes for non-communicable diseases. Due to the increasing prevalence of overweight and obesity, there is a strong focus on dietary overconsumption and energy restriction. Many strategies focus on improving energy balance to achieve successful weight loss. One of the strategies to lower energy intake is refraining from sugars and replacing them with artificial sweeteners, which maintain the palatability without ingesting calories. Nevertheless, the safety and health benefits of artificial sweeteners consumption remain a topic of debate within the scientific community and society at large. Notably, artificial sweeteners are metabolized differently from each other due to their different properties. Therefore, the difference in metabolic fate of artificial sweeteners may underlie conflicting findings that have been reported related to their effects on body weight control, glucose homeostasis, and underlying biological mechanisms. Thus, extrapolation of the metabolic effects of a single artificial sweetener to all artificial sweeteners is not appropriate. Although many rodent studies have assessed the metabolic effects of artificial sweeteners, long-term studies in humans are scarce. The majority of clinical studies performed thus far report no significant effects or beneficial effects of artificial sweeteners on body weight and glycemic control, but it should be emphasized that the study duration of most studies was limited. Clearly, further well-controlled, long-term human studies investigating the effects of different artificial sweeteners and their impact on gut microbiota, body weight regulation and glucose homeostasis, as well as the underlying mechanisms, are warranted.

## Introduction

Diet is among the most important health influencers. Along with globalization and economic growth, a shift in dietary habits has occurred since 1970 (1, 2). Energy intake has increased along with the consumption of animal fat and energy-dense foods, while fiber intake has decreased (2). This dietary shift contributes to the rise of non-communicable diseases, including obesity, type 2 diabetes mellitus (T2DM), cardiovascular disease, and cancer (3–5). A poor diet was found to be the leading risk factor of death and third leading risk factor for disability-adjusted life-years loss in the United States (6). Globally, 11 million deaths and 255 million disability-adjusted life-years were attributable to dietary risk factors in 2017 (7). Due to the increasing trends in overweight and obesity, there is a strong focus on dietary overconsumption and energy restriction. In 2016, there were more than 1.9 billion overweight adults and 650 million obese adults, representing a global prevalence of 13% (8). Beside adults, the prevalence of childhood obesity has also increased dramatically worldwide. Over 340 million children and adolescents (5–19 year of age) were overweight or obese in 2016 (8).

However, obesity and its associated metabolic disorders, including T2DM, cardiovascular disease, and fatty liver disease, are preventable. Many strategies exist to achieve successful weight loss by improving dietary habits and energy balance. However, even more challenging than achieving weight loss is the maintenance of body weight after weight loss (9). The intake of sugar contributes to the overall energy density of diets, thereby promoting obesity (10, 11). In particular the consumption of sugar-sweetened beverages has been associated with cardiometabolic complications, driven by an increased energy intake and obesity (12). Therefore, one common approach to improve energy balance is to refrain from sugars by replacing them with artificial sweeteners. Although the World Health Organization (WHO) recommends free sugar intake of <10% of total energy intake, preferably <5% of total energy intake as a conditional recommendation, a large proportion of the European population appears to exceed this threshold, especially children (13). For instance, 81% of the Dutch population does not fulfill this recommendation as the intake of free sugars equals ~14% of total energy intake in the Netherlands (14).

As artificial sweeteners offer a sweeter taste without calories, the replacement of sugars with these sweeteners seems promising in reducing sugar and energy intake. Meta-analyses of Randomized Controlled Trials (RCTs) have shown that daily energy intake (after 4 or 10 weeks) and sugar intake (after 4 weeks) were lower in healthy, overweight, and obese individuals receiving artificial sweeteners as a replacements of sugars in the diet (15). Sweeteners are classified as natural sweeteners and artificial sweeteners. Artificial sweeteners are further classified as nutritive and non-nutritive sweeteners, depending on whether they contain calories. The nutritive sweeteners include the monosaccharide polyols (e.g., xylitol, mannitol, and sorbitol) and the disaccharide polyols (e.g., lactitol and maltitol). The non-nutritive sweeteners, known as artificial sweeteners, include substances from different chemical classes that are 30–13,000 times sweeter than sucrose (16). Artificial sweeteners are metabolized differently and have different properties, including sweetness intensity, persistence of sweet taste, coating of the teeth, and aftertaste effects (15). Therefore, each sweetener is unique and may affect the perceived taste or use in food applications differently (17). Sweetener consumption is highly prevalent in both adults and children and is expected to increase even more in the near future. In the United states, ~25% of children and >41% of adults consumed artificial sweeteners in 2009–2012, representing a 200% increase in consumption in children and a 54% increase among adults compared to data from 1999 to 2000 (18). Between these decades, a rise in food products containing artificial sweeteners occurred with more than 6,000 new products launched in the United states alone (19). Currently, six different artificial sweeteners are approved by the Food and Drug Administration (FDA) as food additives in the United States, including saccharin, sucralose, aspartame, advantame, acesulfame-potassium, and neotame (20). Furthermore, thaumatin, steviol glycosides, obtained from the leaves of *Stevia* plant, and *Luo Han Guo* fruit extracts have been granted the Generally Recognized as Safe (GRAS) status by the FDA (20, 21). In the European Union, the range of approved artificial sweeteners is broader, as cyclamate, aspartame-acesulfame salt, and neohesperidin dihydrochalcone are also approved by the EU Scientific Committee on Food (22–24). Other artificial sweeteners have not been assessed yet or are declared as unsafe for usage.

Despite the fact that many national authorities have recognized artificial sweeteners as safe and well-tolerated, a lot of controversies about the effects of sweeteners on human health still exist. Whereas, some longitudinal cohort studies show an association between artificial sweeteners consumption and reduced risk of T2DM, overweight and obesity, other observational studies have yielded opposite findings (25–28). Furthermore, longitudinal cohort studies found a positive association between the consumption of artificial sweeteners and the risk of hypertension, stroke, and cardiovascular events (29). Thus, although the use of artificial sweeteners seem promising in assisting weight loss, artificial sweeteners have been linked to a variety of health concerns, including obesity and its related cardiometabolic disturbances (29–31). Importantly, however, it cannot be excluded that the associations found in these observational and prospective cohort studies studies are largely explained by an increase in artificial sweetener intake to compensate for an unhealthy diet or lifestyle in general (reverse causation). The safety and health benefits of artificial sweeteners consumption remain controversial. Considering the rising prevalence of obesity and T2DM along with the increased consumption of artificial sweeteners, it is important to clarify their health benefits and/or harms (18, 32, 33). Therefore, the physiological health effects of artificial sweeteners should be elucidated.

In this review, we provide an overview of the physiological effects of artificial sweeteners on body weight control and glucose homeostasis. Furthermore, the pharmacokinetics of the commonly used artificial sweeteners will be addressed to identify the controversies of the existing evidence surrounding their use. Subsequently, effects of artificial sweeteners on body weight and glycemic control will be discussed.

## Methods

Ample data is available on the effects of artificial sweeteners on body weight and glucose homeostasis. Nevertheless, fewer studies are available reporting the effects of specific artificial sweeteners. A review of the literature was conducted using PubMed databases in the period January–April 2020. The following search terms were used for artificial sweeteners: “artificial sweeteners” OR “non-caloric sweeteners” OR “non-nutritive sweeteners” OR “aspartame” OR “sucralose” OR “acesulfame potassium” OR “acesulfame-K” OR “steviol glycoside” OR “rebaudioside” OR “saccharin.” Different combinations of search terms were used, with and without the artificial sweetener search term, including pharmacokinetics (MeSH terms), body weight (MeSH terms), adiposity (MeSH terms), caloric intake (MeSH terms), sweet taste receptors (MeSH terms), gut-brain axis (MeSH terms), adipogenesis (MeSH terms), microbiota (MeSH terms), short chain fatty acids (MeSH terms), free fatty acid receptors (MeSH terms), energy expenditure (MeSH terms), glucose homeostasis (MeSH terms), insulin secretion (MeSH terms), and inflammation (MeSH terms). Articles written in English language were included. No data restrictions were applied. Reference lists of relevant systematic reviews were screened to identify further relevant citations. Human studies were mainly selected for this review to address the effect of artificial sweeteners on parameters related to body weight or adiposity and glucose homeostasis. In case of limited or lacking human data, rodent studies and *in vitro* studies were also considered. Studies in healthy adults as well as adults living with overweight, obesity or diabetes were included. RCTs (including weight-loss studies), prospective cohort studies, cross-sectional studies, and meta-analyses were included in the literature search. Studies included the use of artificial sweeteners solely, without carbohydrate or caloric content modification, unless specified otherwise. Studies with children (≤18 years), pregnant women, or individuals with acute or chronic diseases other than obesity and diabetes were excluded. Furthermore, studies that did not specify the type of artificial sweetener were excluded. We have identified 5 meta-analyses of RCTs or RCTs studying the effects of specific artificial sweeteners on adiposity and 20 meta-analyses of RCTs or RCTs studying the effects of specific artificial sweeteners on glucose homeostasis as indicated in Tables 1, 2, respectively. Retrieved papers were first screened by title and subsequently by abstract based on the criteria. Full papers were reviewed in case the abstract was insufficient to determine the eligibility. Endnote X8 was used for the management of articles and citations. In total, 164 publications were identified that matched these criteria.

**Table 1:** Characteristics of human studies investigating the effect of specific artificial sweeteners on body weight or adiposity.

**References**	**Study type**	**Duration**	**Participants**	**Dosage artificial sweetener**	**Comparator**	**Adiposity measure**	**Statistical significance**
**Aspartame**							
(34)	Meta-analysis	Acute−16 weeks	Obese, T2DM	162 mg, *ad libitum*, or 500 ml beverage	Sucrose or water	Body weight	N.S.
(34)	Meta-analysis	Acute	Obese, T2DM	162 mg or 500 ml beverage	Sucrose	Body weight	N.S.
**Steviol glycoside**							
(35)	Meta-analysis	90 days−2 years	Healthy, T1DM, T2DM	3.75–1,500 mg/day	Placebo (talcum, maize starch or unspecified)	BMI	N.S.
**Saccharin**							
(36)	RCT	12 weeks	Overweight, obese	1.25–1.75 L/daily	Sucrose	Body weight	N.S.
**Sucralose**							
(37)	RCT	7 days	Healthy	780 mg/day	Placebo (calcium carbonate)	Body weight	N.S.
(38)	RCT	14 days	Healthy	36 mg/day in commercial sachets	Control group	Body weight and BMI	N.S.

*RCT, Randomized Controlled Trial; T2DM, Type 2 Diabetes Mellitus; T1DM, Type 1 Diabetes Mellitus; BMI, body mass index; N.S, non-significant*.

**Table 2:** Characteristics of human studies investigating the effect of specific artificial sweeteners on glucose homeostasis.

**References**	**Study type**	**Duration**	**Participants**	**Dosage artificial sweetener**	**Comparator**	**Measure of glucose homeostasis**	**Statistical significance**
**Aspartame**							
(39)	RCT	Acute	Healthy	169 mg	Water	Glucose levels	N.S
(40)	RCT	Acute	Obese	500 ml beverage	Water	Glucose levels	N.S.
(43)	RCT	Acute	T2DM	400 mg in beverage	Unsweetened flavored beverage	Glucose levels	N.S.
(44)	RCT	Acute	Healthy, overweight	250 mg	Water	Glucose levels	N.S.
(45)	RCT	Acute	Healthy	400 mg	Placebo (corn flour)	Glucose levels	N.S.
(46)	RCT	Acute	Healthy, T2DM	72 mg	Water	Glucose levels	N.S.
(47)	RCT	2 weeks	Healthy	425 mg/day	-	Glucose levels, HbA1c	N.S.
(48)	RCT	2 weeks	Diabetic (not specified)	125 mg/day	-	Glucose levels	N.S.
(49)	RCT	6 weeks	T2DM	163 mg/day	Sucrose	Glucose levels, HbA1c	N.S.
(50)	RCT	18 weeks	T1DM, T2DM	270 mg/day	Placebo (corn starch)	Glucose levels, HbA1c	N.S.
**Steviol glycoside**							
(35)	Meta-analysis	3–6 months	Healthy, T1DM, T2DM	3.75–1,500 mg/day	Placebo (talcum, starch or unspecified)	HbA1c	N.S.
(35)	Meta-analysis	3–24 months	Healthy, T1DM, T2DM	3.75–1,500 mg/day	Placebo (talcum, starch or unspecified)	Glucose levels	N.S.
**Saccharin**							
(43)	RCT	Acute	Healthy, T1DM, T2DM	135 mg in beverage	Unsweetened flavored beverage	Glucose levels	N.S.
**Acesulfame-K**							
(39)	RCT	Acute	Healthy	220 mg	Water	Glucose levels	N.S.
**Sucralose**							
(39)	RCT	Acute	Healthy	62 mg	Water	Glucose levels	N.S.
(51)	RCT	Acute	Healthy	60 mg	Glucose	Glucose levels	N.S.
(52)	RCT	Acute	Healthy	50 ml beverage	Water	Glucose levels	N.S.
(53)	RCT	Acute	Healthy	80 mg infusion	Saline infusion	Glucose levels	N.S.
(42)	RCT	Acute	Healthy	960 mg infusion	Saline infusion	Glucose levels	N.S.
(46)	RCT	Acute	Healthy, T2DM	24 mg	Water	Glucose levels	N.S.
(54)	RCT	10 days	Healthy	60 mg in beverage	-	Insulin sensitivity	N.S.
(54)	RCT	10 days	Healthy	60 mg + maltodextrin	-	Insulin sensitivity	↓, P < 0.043
(47)	RCT	2 weeks	Healthy	0.136 mg/day	-	Insulin sensitivity	N.S.
(38)	RCT	2 weeks	Healthy	36 mg/day + maltodextrin/ dextrose	Control group	Insulin sensitivity	−17.7%, P < 0.04
(55)	RCT	13 weeks	T2DM	667 mg/day	Placebo (cellulose)	HbA1c	N.S.

*Acesulfame-K, acesulfame potassium; RCT, Randomized Controlled Trial; T2DM, Type 2 Diabetes Mellitus; T1DM, Type 1 Diabetes Mellitus; HbA1c, glycated hemoglobin; N.S, non-significant*.

## Pharmacokinetics

**Figure 1:**
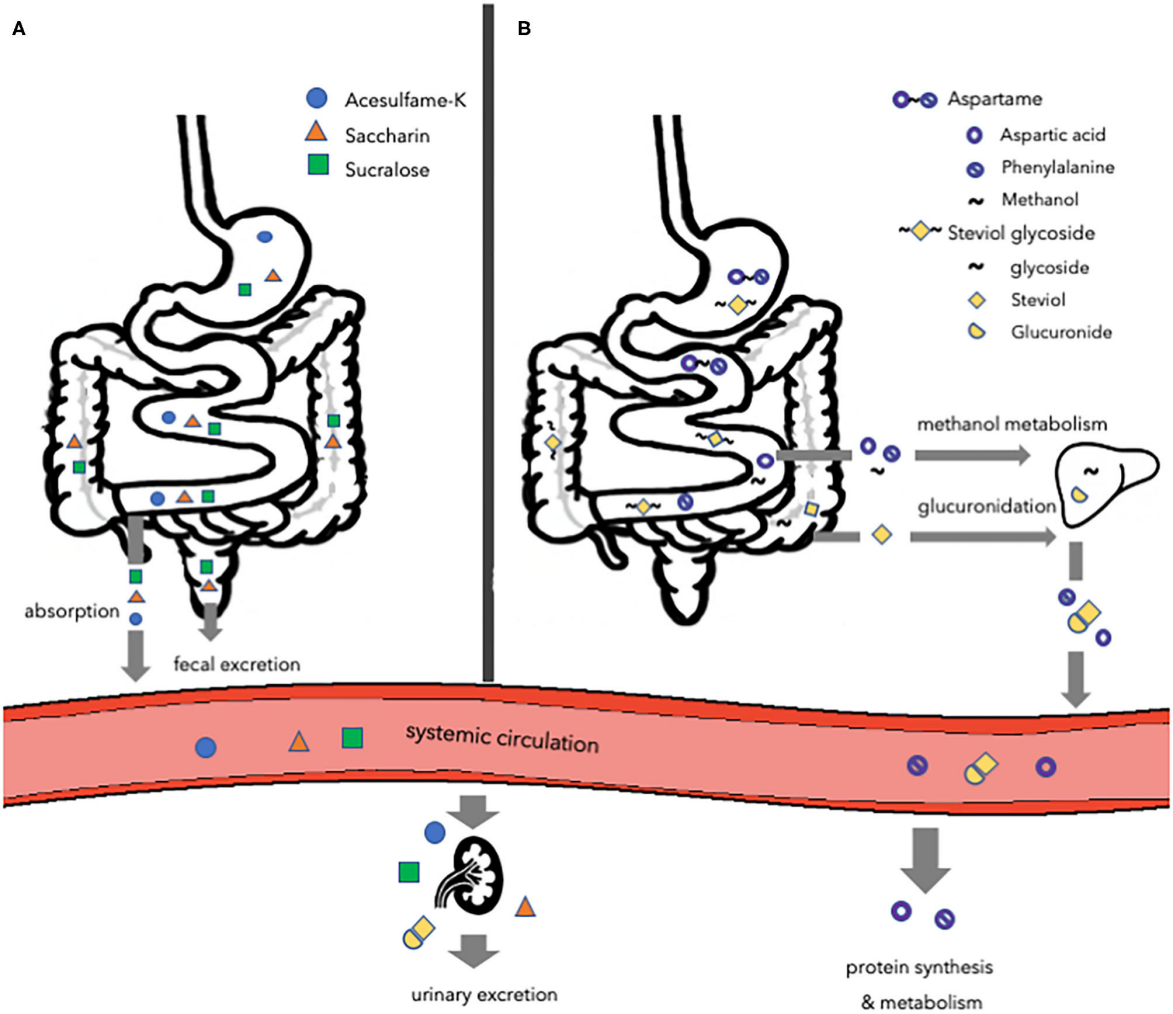
Overview of the major routes of absorption, digestion, metabolism, and excretion of different types of artificial sweeteners. **(A)** Acesulfame-K, saccharin, and sucralose. Acesulfame-K is completely absorbed into the systemic circulation to be excreted in the urine via the kidneys. The majority of saccharin is absorbed and distributed, while the remaining amount passes the gastrointestinal tract to be eliminated in the feces. Most of the sucralose passes the gastrointestinal tract to be eliminated in the feces, while a small amount is directed toward the kidneys to be excreted in the urine. **(B)** Aspartame and steviol glycoside. Aspartame is digested in the small intestine and the hydrolyzed components are absorbed and metabolized following their normal metabolic pathways. Steviol glycoside is fermented by the gut microbiota to form steviol, which is absorbed into the liver and excreted in the urine. Acesulfame-K, acesulfame potassium.

To determine safety of artificial sweeteners the FDA considers probable intake, cumulative effects from all uses, and toxicological data in animals. The European Food Safety Authority (EFSA) evaluates and confirms that the intake of artificial sweeteners, within the acceptable daily intake (ADI), does not cause cancer or other health-related problems, and are therefore safe for human consumption (56, 57). Although authorities consider artificial sweeteners as safe as they do not pose any health-related problems, when consumed within the ADI, no specific safety claims have been made about the effects of sweeteners on non-communicable diseases, such as obesity and T2DM. Despite the fact that several artificial sweeteners are tested for pharmacological and toxicological aspects, the concerns about the effects of unmetabolized compounds on non-communicable diseases still exist. Artificial sweeteners have distinct structures and are metabolized differently as some but not all are digested or fermented (Figure 1). The most common artificial sweeteners such as acesulfame potassium, saccharin, aspartame, sucralose, and steviol glycoside will be discussed in the present review.

### Acesulfame Potassium

Acesulfame potassium (acesulfame-K) (6-Methyl-1,2,3-oxathiazin-4(3H)-one 2,2-dioxide), belonging into the oxathiazinodioxide class of chemicals, is a white crystalline powder and is ~200 times sweeter compared to sucrose (58, 59). Due to the higher intensity and the longer persistence of the sweetness, acesulfame-K is used in a wide range of products, mainly soft drinks. Although this sweetener contains potassium, its intake does not influence systemic potassium levels (60). Acesulfame-K is not metabolized by the body (61). Following ingestion, acesulfame-K is completely absorbed into the systemic circulation and distributed (58, 62) (Figure 1). The absorption of acesulfame-K is very rapid, thereby making it unlikely that it will reach the lower gastrointestinal (GI) tract to impact the gut microbiota upon administration of a normal ADI-dosage (63). Within 24 h after ingestion, acesulfame-K is primarily excreted via the kidneys into the urine (>99%), with <1% excreted in feces (58, 62).

### Saccharin

Saccharin (1,1-dioxo-1,2-benzothiazol-3-one) is available in three different forms: in acid form, or bound to sodium or calcium (64). The most common form is sodium salt due to its high solubility and stability. Saccharin is ~300 times sweeter than sucrose (62, 64). Similarly to acesulfame-K, saccharin is not metabolized by the body (65). Therefore, the FDA considers saccharin as safe (20). After ingestion of saccharin, ~85–95% is absorbed and bound to plasma proteins to be distributed via blood (58) (Figure 1). Thereupon, the saccharin is excreted in the urine, while the remaining 5–15% passes through the GI-tract entirely to be eliminated in the feces unchanged (58, 66). Therefore, a fraction of saccharin that is not immediately absorbed is able to affect the gut microbiota composition (58).

### Aspartame

Aspartame ((3S)-3-amino-4-[[(2S)-1-methoxy-1-oxo-3-phenylpropan-2-yl]amino]-4-oxobutanoic acid) is approximately 200 times sweeter than sucrose (58). In contrast to other artificial sweeteners, aspartame contains 4 calories per gram. Nevertheless, due to the sweetening intensity, only a small amount of aspartame is used in products to achieve sweetness. Therefore, few calories are derived from aspartame in sweetener products. Upon ingestion, aspartame is broken down in the small intestine by esterases and peptidases to aspartic acid, phenylalanine, and methanol (16, 67) (Figure 1). Only the hydrolyzed components are absorbed into the circulation and metabolized following their normal metabolic pathways (68). Methanol is metabolized in the liver, while aspartate acid and phenylalanine enter the free amino acid pool. Thereupon, the components are taken up by peripheral tissues, utilized for protein synthesis and metabolism, and excreted. Aspartame does not accumulate in the body as it is rapidly digested (57). Neither aspartame nor its components reach the colon. Therefore, aspartame is not able to affect the gut microbiota (58, 69).

### Sucralose

Sucralose (2R,3R,4R,5R,6R)-2-[(2R,3S,4S,5S)-2,5-bis (chloromethyl)-3,4-dihydroxyoxolan-2-yl]oxy-5-chloro-6-(hydroxymethyl)oxane-3,4-diol) is very similar to sucrose in structure. However, the three hydroxyl groups attached to the sucrose molecule are replaced by chlorine atoms, thereby changing the confirmation of the molecule, to form sucralose (58). Thus, glycosidic enzymes are unable to recognize and digest sucralose. Although sucralose is made from sugar, it provides no calories as it is not digested in the body (16, 70). Sucralose is 600 times sweeter compared to sucrose. Most of the sucralose passes through the GI tract entirely to be directly eliminated in the feces, whereas a small amount (11–27%) is absorbed and is directed toward the kidneys to be excreted in the urine (71) (Figure 1). Nevertheless, sucralose was found to be non-nutritive to bacteria and resistant to fermentation, while affecting microbiota through bacteriostatic effects (72).

### Steviol Glycoside

Steviol glycosides (13-Hydroxykaur-16-en-18-oic acid) are the chemical compounds responsible for the sweet taste and can be found on the leaves of the South American plant *Stevia rebaudiana* (73). Steviol glycosides are ~100 to 300 times sweeter compared to sucrose (73). Steviol glycosides cannot be hydrolyzed by the digestive enzymes and acids present in the upper GI tract (58, 74). Nevertheless, the microbiota in the colon, primarily Bacteroides, is able to degrade steviol glycosides (75). Therefore, steviol glycosides are able to modulate the gut microbiota as they encounter it directly. Steviol glycoside is degraded by cleavage of the glycoside linkage, thereby forming steviol, steviolbioside, and glucose (76–78) (Figure 1). In turn, steviolbioside will be converted to steviol (78). The formed glucose is either utilized by colonic bacteria or absorbed, metabolized, and excreted into the expired air as carbon dioxide and water, while steviol is absorbed and enters the liver via the portal vein (79, 80). Nonetheless, the entry of steviol into the portal vein is slow due to the slow metabolization by the colonic bacteria, depending on the species (81). In the liver, steviol is glucoronidated and excreted into the urine (82, 83).

### Body Weight and Adiposity

**Figure 2:**
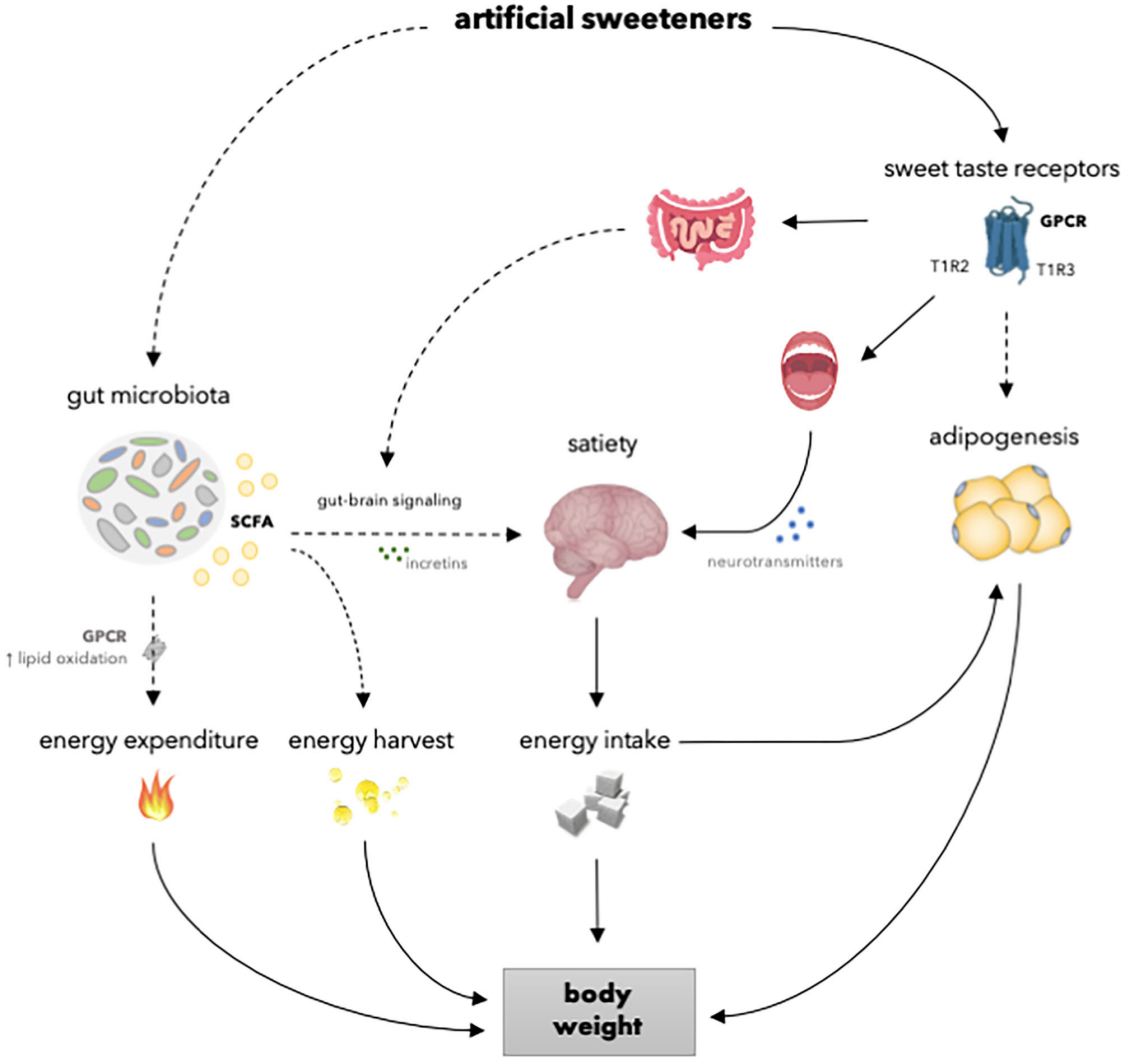
Overview of the mechanisms of how artificial sweeteners may affect physiological processes involved in body weight regulation. Artificial sweeteners interact with T1R-family of sweet-taste receptors in the oral cavity and gastrointestinal tract, thereby able to affect satiety and, in turn, energy intake and body weight. However, *in vivo* studies have shown no effect of artificial sweeteners on the secretion of incretins. Furthermore, several artificial sweeteners may reach the adipose tissue to interact with T1R-family of sweet-taste receptors and affect adipogenesis and, in turn, body weight. Moreover, several artificial sweeteners are able to induce gut microbiota alterations. Thereupon, SCFA production is enhanced. It can be speculated that SCFA may, in turn, increase energy expenditure due to enhanced lipid oxidation and affect satiety by modulating gut-brain signaling via incretins. Dashed lines indicate that the effect is dependent on type of artificial sweetener and/or that results are inconsistent or hypothetical. SCFA, short chain fatty acids; GPCR; G-protein coupled receptor; T1R2, taste receptor type 1 member 2; T1R3, taste receptor type 1 member 3.

An increased body weight and adiposity develop under conditions of a positive energy balance. The regulation of energy balance is a complex process that involves homeostatic regulation of energy intake and energy expenditure. Although artificial sweeteners are as sweet or even sweeter than natural sugars, the caloric content and the metabolism routes are different. Therefore, it is likely that artificial sweeteners may affect energy balance, and thus body weight, differently compared to natural sugars via underlying physiological processes comprising the gut microbiota, the reward-system, and adipogenesis (Figure 2). Considering the increase in the prevalence of overweight and obesity and the rising interest in losing weight, preventing weight gain and maintaining weight loss, it is important to elucidate the effects of artificial sweeteners on body weight control. Meta-analysis, based on RCTs, showed that there is no significant difference in body weight change between overweight and lean individuals consuming artificial sweeteners compared to those receiving sugars or cellulose as placebo for <6 months (15). Furthermore, Azad et al. (29) reported no significant effects of artificial sweeteners on weight change compared to sugar or water in people living with obesity, based on meta-analysis of long-term RCTs (≥6 months). Interestingly, however, other meta-analysis of RCTs (4 weeks to 40 months) showed that the intake of artificial sweeteners resulted in reduced body weight in overweight and lean individuals compared to sugar or water (84). Notably, however, this meta-analysis included 4 out of 12 intervention studies carried out in the context of a weight loss program (84). Nevertheless, these findings strongly suggest that artificial sweeteners may have neutral or beneficial effects on long-term body weight control.

Considering specific types of artificial sweeteners, meta-analyses, based on RCTs, showed no effect of aspartame consumption on body weight compared to sugar or water in individuals with either obesity or T2DM (34) (Table 1). Only studies wherein aspartame was evaluated alone were included in the meta-analyses to clarify the specific effects of aspartame without interference of results obtained due to the consumption of other sweeteners. However, large heterogeneity was found due to different treatment patterns for aspartame and sugar or water. Similarly, meta-analysis, based on RCTs, showed no effect of steviol glycoside consumption on BMI compared to talcum, maize starch, or unspecified matching placebo in healthy individuals and patients with diabetes (35). Additionally, subgroup analyses showed no significant effect of steviol glycoside on BMI in either healthy individuals and patients with diabetes. The results indicate that these artificial sweeteners do not affect body weight. However, the effects of acesulfame-K and saccharin can still be debated, as there is no consistent evidence, and meta-analyses are lacking. More specifically, one study that used the ADI-dosage for human consumption (15 mg/kg/day) showed no effect on body weight in mice after 8 weeks of acesulfame-K consumption, while another study shows an increase in body weight by exceeding the ADI more than 2-fold (37.5 mg/kg/day) after 4 weeks in mice (85, 86). Furthermore, saccharin consumption was found to increase body weight in mice compared to water, sucrose or glucose, whereas other studies in rodents have shown reduced or unchanged body weight compared to mice receiving water, glucose, fructose or sucrose (87–94). However, the absorption of saccharin is lower in rodents compared to humans due to a relative higher stomach pH in rodents (92). Furthermore, differences in perception of sweetness for individual artificial sweeteners exist between different rodent species and strains (95). Therefore, perception and post-ingestive responses of rodents might differ from humans. Nevertheless, human data on the effect of acesulfame-K on body weight is currently lacking. Moreover, human data on the effect of saccharin on body weight is scarce with only one study showing no significant effects on body weight after 12 weeks of saccharin consumption compared to sucrose in overweight and obese individuals (36). Moreover, sucralose consumption has been reported to have no effect on body weight in mice compared to water, and in human studies compared to placebo (calcium carbonate) or control (no-intervention) (37, 38, 85, 88, 96). Notably, contradictory results from rodent studies for the effect on body weight exist only for acesulfame-K and saccharin, which are largely or entirely absorbed in their intact form, thereby being able to reach the peripheral tissues. Consistently, rodent and human studies found no effect of sucralose on body weight as only a small amount is absorbed in its intact form, thereby reaching the microbiota in a larger amount compared to acesulfame-K and saccharin (37, 38, 85, 88, 96). As artificial sweeteners have different metabolic fates, differences in physiological effects affecting energy balance and adiposity should be elucidated.

## The Interaction Between Artificial Sweeteners, Reward, and Adiposity

### Reward

As artificial sweeteners contain no or low amounts of calories, one might expect that these sweeteners may contribute to lower energy intake and thus body weight reduction. Nevertheless, controversies exist whether artificial sweeteners affect appetite, hunger, and eating behavior, and if these effects are beneficial or not. One driving aspect in eating behavior is the reward of food. The reward system plays an important role in regulating energy intake, and can be divided into sensory and post-ingestive reward (19, 97). After ingestion of either natural sugars or artificial sweeteners, gustatory information is perceived by sweet taste receptors, which are heterotrimeric G-protein coupled receptors (GPR) consisting of two subunits, namely taste receptor type 1 member 2 (T1R2) and 3 (T1R3) (98, 99). The sweet taste receptors are located in taste buds in the oral cavity and outside the oral cavity, including the intestine and pancreatic β-cells (100). The binding sites of sweet taste receptors are different for artificial sweeteners and natural sugars (101). Upon interaction of sweet compounds to the sweet receptor T1R2/T1R3, the heterotrimeric G protein, α-gustducin, is activated (102). As a result, the subunits Gβγ are dissociated and can interact with phospholipase Cβ2 (PLC-β2), which in turn increases production of inositol 1,4,5-triphosphate and diacylglycerol (103). Consequently, the transient receptor potential cation channel subfamily M member 5 is activated, thereby increasing intracellular calcium and neurotransmitter release (104–106). As artificial sweeteners and natural sugars bind differently to the sweet taste receptors, the gustatory branch is activated differently as well (19, 101). Thereupon, artificial sweeteners may generate weaker signals that are sent to areas involved in reward and satisfaction, as consistently demonstrated by using functional Magnetic Resonance Imaging (fMRI) in several randomized cross-over trials (107, 108).

Likewise, the ingestion of artificial sweeteners induces a signaling cascade outside of the oral cavity. Within the GI tract, sweet taste receptors are primarily located on enteroendocrine L- and K-cells (104). The signal transduction pathway is similar as in cells present in the oral cavity. Upon ligand binding of natural sugars to sweet taste receptors, enteroendocrine L-cells secrete glucagon-like peptide-1 (GLP-1) and peptide YY (PYY), whereas K-cells secrete glucose-dependent insulinotropic peptide (GIP) (100). These hormones are able to cross the semi-permeable blood-brain barrier, thereby reaching the hypothalamus and affecting food intake by reducing appetite and increasing satiety (41). However, artificial sweeteners may not be potent secretagogues for GLP-1, PYY, and GIP to the same extent *in vivo* as natural sugars, since the secretion is nutrient-dependent (39, 109, 110). For instance, aspartame is digested and absorbed before reaching the lower GI tract to bind to the sweet taste receptors. Acesulfame-K, sucralose, steviol glycoside, and saccharin pass through the lower GI tract to be absorbed, digested or eliminated directly. Consistently, mice studies and human crossover trials in lean and obese individuals have shown no significant effects of artificial sweeteners on incretin secretion (39, 40, 42, 51–53, 111, 112). In addition to the lack of an effect on incretin secretion, two human crossover studies showed no effect on appetite upon sucralose or aspartame-sweetened diet coke consumption in healthy and obese individuals (40, 52). Furthermore, randomized cross-over trials showed weaker reward and satisfaction signals upon aspartame or sucralose ingestion in healthy individuals, thereby suggesting that caloric intake is required in evoking a hypothalamic response (107, 108). Therefore, it has been suggested that artificial sweeteners do not activate the food reward pathways in the same way as natural sugars. The elimination of the post-ingestive reward holds true for non-caloric artificial sweeteners, whereas the intake of artificial sweeteners in the presence of carbohydrates may elicit post-ingestive incretin responses, as demonstrated using sucralose-sweetened beverages (54). Based on the above, it can be postulated that artificial sweeteners solely offer less reward compared to natural sugars, although it should be emphasized that the differences in reward response has not been shown in the context of a whole-meal approach or diets, where sugar was replaced by artificial sweeteners.

### Energy Intake

The lack in complete satisfaction may drive the assumption that artificial sweeteners fuel food seeking behavior, thereby contributing to increased or no differences in energy intake. However, less satisfaction does not necessarily translate into compensatory (excess) energy intake (113–116). RCTs have shown that the reduced caloric intake by replacing natural sugars with artificial sweeteners is not completely compensated (117, 118). As a result, energy intake after the use of artificial sweeteners is still lower compared to natural sugars, even after putative compensatory energy intake. Therefore, the compensatory energy intake does not seem to pose a threat to weight gain and may aid in weight loss (maintenance). Furthermore, meta-analysis of acute RCTs (≤1 day) showed that artificial sweeteners decrease energy intake in comparison to caloric sweeteners in overweight and lean individuals, whereas no difference was found in comparison with water (84). In a meta-analysis of long-term RCTs (4 weeks to 40 months), artificial sweeteners were found to decrease energy intake compared to caloric sweeteners or water (84). Similarly, a meta-analysis including RCTs with a study duration of 4–10 weeks showed reduced energy and sugar intake in lean and overweight individuals consuming artificial sweeteners compared to those receiving sugar (15). Taken together, these findings suggest that compensatory energy intake during consumption of artificial sweeteners does not seem to occur in the short- and long-term, or at least does not completely compensate for the reduced caloric intake compared to sugar intake.

### Adipogenesis

Sweet taste receptors are expressed in many organs, including adipose tissue (119). Not all artificial sweeteners will reach the adipose tissue as some are not absorbed into the systemic circulation. The sweet taste-sensing receptor in adipose tissue differs in comparison to the receptors in sweet taste buds or in the GI tract. In adipocytes, the expression of T1R3 was found to be higher than T1R2, suggesting that a higher percentage of T1R3 is present as a homomer (120). Nevertheless, increased adipogenesis and reduced lipolysis were found, independent of T1R2 and T1R3, upon *in vitro* stimulation of adipocytes with saccharin (119). It has been suggested that saccharin act on a protein kinase A-mediated mechanism downstream of cyclic adenosine monophosphate (cAMP). Consequently, hormone sensitive lipase (HSL) phosphorylation is reduced by regulating HSL phosphatase, thereby inhibiting lipolysis (119). Likewise, acesulfame-K was found to stimulate adipogenesis (119). However, the active concentrations of saccharin and acesulfame- K in adipocytes (4.5 mM) were higher than expected to be observed in humans as bolus oral doses of maximum daily intake of saccharin, for instance, results in peak plasma concentrations of ~75 μm (119). Similarly, other *in vitro* studies in human mesenchymal stem cells showed increased fat accumulation and upregulation of genes involved in adipogenesis upon stimulation with a higher sucralose concentration (0.45 or 4.5 mM) (121). Notably, as discussed earlier, contradictory results regarding body weight were found for acesulfame-K and saccharin. Thus, since these artificial sweeteners are largely or entirely absorbed, it could be argued that they reach the adipose tissue and may impact adipogenesis. Nevertheless, Masubuchi et al. (120) showed reduced adipogenesis in *3T3-L1* cells upon stimulation with saccharin or sucralose (20 mM) by activation of adenylate cyclase-cAMP signaling pathway. Along with cAMP-dependent pro-adipogenic signals, cAMP-independent anti-adipogenic signals are generated, which may dominate the formal signal to inhibit adipogenesis (120). Hence, studies investigating the role of artificial sweeteners and peripheral sweet taste receptors are scarce, and existing *in vitro* studies examining the effects of artificial sweeteners on adipogenesis provide inconsistent results (119–121).

## The Interaction Between Artificial Sweeteners, Gut Microbiota, and Energy Balance

### Alterations in Gut Microbiota

Gut microbiota and the produced microbial fermentation products are key to many aspects of human health (122). Besides the involvement of fermenting indigestible food components, gut microbiota seems closely linked to metabolism, energy balance, and the immune system (123). An important modifying factor influencing the composition of the microbiota, and thereby the overall health, is diet (124). Artificial sweeteners may alter the gut microbiota composition, evidenced by increased gut microbiota dysbiosis and an increased Firmicutes:Bacteroidetes ratio in a cross-sectional study with morbidly obese individuals (125). Moreover, another cross-sectional study showed no association between aspartame or acesulfame-K consumption and bacteria abundance profiles or predicted gene function (126). However, bacterial diversity differed between aspartame or acesulfame-K consumers and non-consumers (126). Furthermore, Suez et al. (89) demonstrated that artificial sweeteners are able to induce glucose intolerance in mice and distinct human subsets by altering the gut microbiome. Supplementation of saccharin (5 mg/kg/d) for 1 week induced an elevated glycemic response after an oral glucose load, which was associated with microbiome alterations in a small group of study participants clustered as “responders” (*n* = 4), while no response was found in the other individuals (“non-responders”, *n* = 3) (89). The poor glycemic response in the “responders” was replicated in mice upon fecal transplantation. Similarly to the above mentioned cross-sectional study (126), the microbiome composition between the “responders” and “non-responders” were already distinct prior to saccharin exposure, thereby suggesting that humans feature an unique response to artificial sweeteners and that the gut microbiome may serve as a predictor for the susceptibility (89). Nevertheless, in the latter study there was no placebo group in the short-term intervention study and the number of individuals was small, indicating that replication of these findings is required. Overall, human trials investigating the effect of artificial sweeteners on gut microbiota are scarce.

Regarding rodent studies, an increased Firmicutes:Bacteroidetes ratio, resembling that of obese individuals, was found in mice after 11 weeks of saccharin consumption (89). Consistently, modulation of the gut microbiota was found in other rodent studies upon saccharin consumption, as a minor fraction of saccharin is not absorbed and will concentrate in the colon (96, 127). Besides saccharin, sucralose was consistently found to affect microbiota in mice as it accumulates in the colon (85, 88, 96). However, contradictory results regarding the effect of acesulfame-K on gut microbiota composition have also been found in rodents (85, 86). This discrepancy is at least partly explained by the difference of administered dosage. More specifically, one study that used the ADI-dosage for human consumption (15 mg/kg/day) showed no effect on microbiota composition in mice after 8 weeks consumption, while another study that applied a dosage that exceeds the ADI more than 2-fold (37.5 mg/kg/day), showed an increase in Bacteroides and Firmicutes after 4 weeks consumption in mice (69, 85, 86). Since the absorption of acesulfame-K is very rapid, it is unlikely that it will reach the lower GI tract upon administration of a normal ADI-dosage (63). Regarding other artificial sweeteners, aspartame does not affect the gut microbiota, since it is digested and broken down into residual components before entering the lower GI tract (58). Whereas, steviol glycoside encounters the microbiota directly in order to be fermented. Controversial results exist between *in vivo* and *in vitro* studies using human feces as well as *E.coli* cell lines. *In vitro* fermentation studies using human feces showed no effect of steviol glycoside on microbiota composition (75, 128). Other *in vitro* studies using *E.coli* cell lines showed selective growth inhibition upon steviol glycoside stimulation, or little or no effect on bacterial growth (96, 129). Nevertheless, the consumption of steviol glycoside (2–3 mg/kg) was found to alter gut microbiota composition in mice after 9 weeks (130).

As gut microbiota is closely linked to many aspects of health, changes in microbiota composition may lead to negative alterations in metabolic homeostasis. Suez et al. (89) showed an increase in the glycan degradation pathway, along with an increased Firmicutes:Bacteroidetes ratio, in mice after 11 weeks of saccharin consumption. As a result, glycans are fermented to form short chain fatty acids (SCFA), including acetate and propionate (89, 131). In addition, sucralose was found to increase cecal propionate levels in mice after 8 weeks of consumption (132). In contrast, acesulfame-K consumption did not affect SCFA levels in mice following 8 weeks of consumption upon normal ADI-dosage (85). Furthermore, steviol glycoside was found to increase SCFA after 9 weeks of steviol glycoside consumption in rodents and in studies using an *in vitro* model of the human colon (GIS1) (130, 133). The increase in SCFA levels may be an indicator of enhanced energy harvest, as the capacity to extract energy has been suggested to be increased as result of artificial sweetener consumption. Butyrate, particularly, serves as an energy supply for ~60–70% for colonocytes and gut epithelial cells (134, 135). Whereas, acetate mainly contributes to lipogenesis in the cytosol of hepatocytes and adipocytes or can be oxidized in skeletal muscle (136, 137). In addition, propionate serves as a precursor for gluconeogenesis, lipogenesis, and protein synthesis (89, 138, 139). However, the significance of energy harvest in humans is still unclear, and increased SCFA concentrations have merely been associated with beneficial health effects in humans (140).

### Gut-Brain Signaling

In the small intestine, propionate is able to bind to GPR43 and GPR41, free fatty acid receptors (FFAR) 2 and 3, respectively, in the enteroendocrine L-cells (141). Upon binding to the receptors, the secretion of GLP-1 and PYY is stimulated (142). Mice lacking FFAR2 or FFAR3 were found to have reduced SCFA-triggered GLP-1 secretion *in vitro* and *in vivo* (143). Furthermore, we have recently performed a double-blind, crossover study, showing increased PYY concentration upon acute colonic administration of mixtures of acetate, propionate, and butyrate in overweight or obese men (144). Therefore, it is tempting to speculate that artificial sweeteners, that are able to modulate gut microbiota, are able to affect gut-brain signaling, via increased SCFA production. Besides gut-brain signaling, SCFA are found to affect appetite regulation and leptin secretion, as described more extensively elsewhere (140). Nevertheless, human studies investigating the effect of artificial sweeteners on hunger-satiety cycle, via SCFA, are currently lacking.

### Energy Expenditure

Besides affecting the hunger-satiety cycle, SCFA may modulate body weight control by influencing energy expenditure. Our recently performed double-blind, crossover study, showed increased lipid oxidation, and thus energy expenditure, upon acute colonic infusions of SCFA in overweight or obese men (144). Consistently, mice studies have shown increased lipid oxidation by increasing sympathetic activity in brown adipose tissue, via gut-neural signaling, upon SCFA administration (145–147). However, the relevance of brown adipose tissue in body weight regulation in humans seems less evident, as it may only contribute to a very minor extent to energy expenditure (148). Acetate and butyrate were found to enhance lipid oxidation in mice studies and *in vitro* studies using bovine hepatocytes, possibly mediated via GPR41 and GPR43 (140, 141, 149–152). Nevertheless, *in vivo* studies found no effect on energy expenditure in mice after 40 weeks of acesulfame-K exposure or 5 weeks of saccharin exposure (89, 153). Similar to findings in liver, SCFA were found to enhance lipid oxidation in skeletal muscle as shown in rodents and C2C12 myotubes (154–156). However, human data regarding the effects of SCFA on tissue metabolism are currently lacking. Moreover, human evidence of the effects of artificial sweeteners on microbiota alterations, and subsequently SCFA production, are very limited. Thus, although it is tempting to speculate that artificial sweeteners may affect energy expenditure through altered SCFA production in the gut, further studies are needed to investigate this.

Importantly, the putative beneficial effects of the intake of artificial sweeteners, by SCFA production, are mainly based on studies in rodents. Furthermore, no difference in energy expenditure, using ventilated-hood and 24 h whole body indirect calorimetry, was found upon sucralose consumption in acute studies and long-term (10 weeks) RCTs, whereas lipid oxidation was enhanced and carbohydrate oxidation was decreased compared to sucrose in normal weight and overweight individuals (157, 158). Moreover, no changes in energy expenditure, estimated based on accelerometry, were observed upon saccharin-, aspartame-, sucralose-, or steviol glycoside-sweetened beverage consumption for 12 weeks compared to sucrose in overweight or obese individuals (36). These findings may imply that a reduction in energy intake rather than an increase in energy expenditure may contribute to the beneficial effects of sucralose on body weight control.

## Glucose Homeostasis

**Figure 3:**
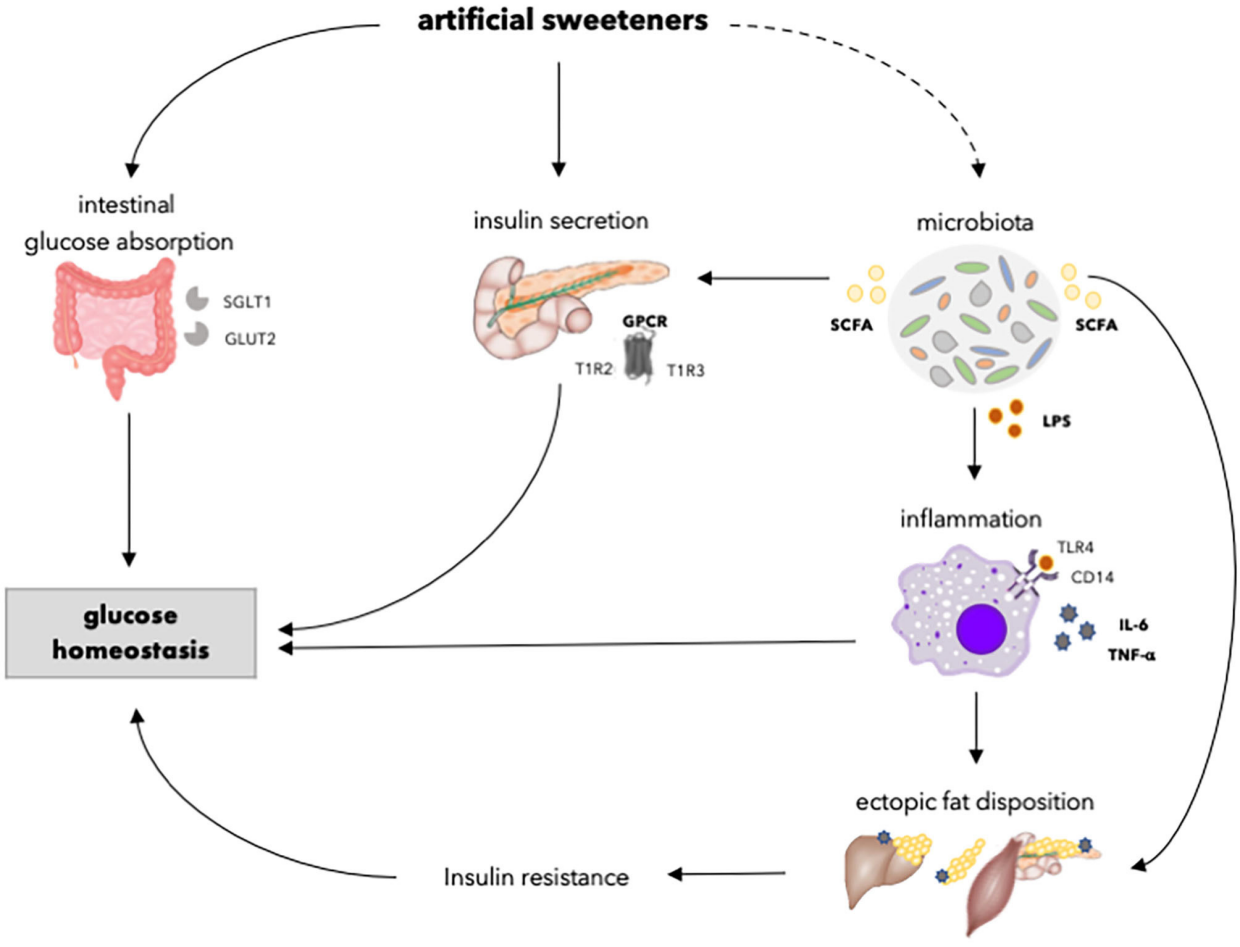
Overview of the effects of artificial sweeteners on physiological processes involved in glucose homeostasis. Artificial sweeteners may enhance intestinal glucose absorption by upregulating SGLT1 and GLUT2. Furthermore, artificial sweeteners affect insulin secretory capacity by interacting with GPCR. Moreover, the artificial sweetener-induced gut microbiota dysbiosis, in turn, may affect insulin secretion via the enhancement of SCFA. Upon dysbiosis, LPS levels may increase, and endotoxemia and chronic inflammation occurs, which might affect ectopic fat accumulation and insulin resistance. Dashed lines indicate that the effect is dependent on type of artificial sweetener. SGLT1, sodium glucose transporter 1; GLUT2, glucose transporter 2; GPCR, G-protein coupled receptor; T1R2, taste receptor type 1 member 2; T1R3, taste receptor type 1 member 3; SCFA, short chain fatty acids; LPS, lipopolysaccharide; TLR4, toll-like receptor 4; CD14, cluster of differentiation antigen 14.

Besides potentially affecting body weight control, artificial sweeteners may also affect glycemic control, since glucose absorption may be reduced upon replacement of available carbohydrates. However, this does not necessarily translate into an improved glucose homeostasis, since alterations in intestinal glucose transport and absorption, insulin resistance, and reduced insulin secretory capacity by artificial sweeteners may contribute to impaired glucose homeostasis (Figure 3). However, the results of systemic reviews and meta-analysis that have been performed to investigate the relationship between artificial sweetener intake and glucose homeostasis or risk of T2DM are controversial. Daher et al. (159) reported that the majority of systemic reviews and meta-analysis, based on RCTs or prospective cohort studies in healthy individuals yielded no conclusive evidence that artificial sweeteners increase the risk for T2DM. Other intervention studies in healthy individuals and patients with diabetes showed no significant effect of artificial sweeteners on glucose homeostasis (glucose and insulin levels) (159). On the other hand, systematic reviews and meta-analysis, based on prospective cohort studies in healthy individuals, showed a positive association between artificial sweetener intake and the incidence of T2DM, independent of adiposity (although attenuated after adjustment for BMI) (159). However, the evidence for a relationship between artificial sweeteners and T2DM is based on prospective cohort studies using only baseline exposure and may be caused by reverse causation. Hence, evidence from systematic and meta-analysis does not consistently show that artificial sweeteners reduce the risk of T2DM in humans.

Considering specific types of artificial sweeteners, glucose homeostasis seems to be unaffected by aspartame and steviol glycoside. No significant effect on glucose levels and glycated hemoglobin (HbA1c) levels were found after acute or long-term aspartame consumption (39, 40, 43–50) (Table 2). Similarly, a meta-analysis of long-term RCTs showed no effect of steviol glycoside on glucose levels and HbA1c levels in healthy individuals and patients with diabetes (35). Regarding other artificial sweeteners, glucose levels were not found to be affected by acute saccharin consumption in healthy individuals and patients with diabetes, and acute acesulfame-K consumption in healthy individuals (39, 43). In addition, mice studies found no effect on glucose tolerance upon acesulfame-K consumption (153). Nevertheless, data from rodent studies on saccharin consumption remain controversial, as one study showed an increase in glucose tolerance after 11 weeks of commercial saccharin added to drinking water, whereas another study found no effect after 7 weeks of pure saccharin added to drinking water (89, 160). However, the discrepancies may be explained by differences in caloric content of the drinking water, as the study showing increased glucose tolerance used a commercial sweetener (Sucrazit), consisting out of 95% glucose and 5% saccharin, whereas the other study showing no effect used pure saccharin (89, 160). More specifically, one study that used the ADI-dosage for human consumption (15 mg/kg/day) showed no effect on body weight in mice after 8 weeks of acesulfame-K consumption, while another study shows the opposite by exceeding the ADI more than 2-fold (37.5 mg/kg/day) after 4 weeks in mice (85, 86). Furthermore, glucose and HbA1c levels were not affected by acute or long-term sucralose consumption in healthy individuals and patients with diabetes (39, 42, 46, 51–53, 55). Remarkedly, short-term sucralose consumption alone showed no effect on insulin sensitivity in healthy individuals, whereas sucralose-sweetened beverages, containing carbohydrates, or sucralose sachets added to carbohydrate-containing beverages or meals, decreased insulin sensitivity in healthy individuals (38, 47, 54). Therefore, it has been suggested that sucralose may impair glucose metabolism only when co-ingested with carbohydrates. The role of artificial sweeteners in enhancing intestinal glucose absorption, thereby perturbating glucose homeostasis in the presence of carbohydrate content, can be speculated (as discussed below). The discrepancies of the effects of artificial sweeteners on glucose homeostasis may be explained by the difference in types of artificial sweeteners and the intake of artificial sweeteners solely or in combination with carbohydrates. Nevertheless, more human studies are needed to confirm these findings, and assess whether these putative effects on glucose homeostasis can be translated to a situation where artificial sweeteners are consumed as part of the diet with other dietary components.

### Intestinal Glucose Absorption

The GI tract plays a major role in the regulation of glucose homeostasis. As artificial sweeteners may impact gut microbiota and function, they are able to alter intestinal glucose absorption and thus postprandial glucose levels. Upon ingestion of carbohydrates, glucose is largely absorbed across the enterocytes of the intestinal wall via sodium-glucose cotransporter-1 (SGLT1) on the apical membrane and the passive glucose transporter 2 (GLUT2) on the basolateral membrane (106). The sweet taste receptors located in the GI tract serve as glucose sensors to adapt dietary glucose concentrations (161). Upon binding of glucose to the sweet taste receptors, the secretion of GLP-1, GLP-2, and GIP is enhanced, which in turn increases the expression of GLUT2 (162, 163). However, artificial sweeteners alone seem not able to elicit the same effects as natural sugars *in vivo* due to lack of caloric content, as discussed earlier. Nevertheless, SGLT1 was found to be upregulated by sucralose, acesulfame-K, and saccharin in wild-type mice, but not in mice lacking T1R3 or α-gustducin (161). This was not found for aspartame, as mice do not sense it as sweet (161). In addition, sucralose, acesulfame-K, and saccharin were found to increase GLUT2 insertion into the apical membrane, thereby increasing the rate of intestinal glucose absorption in mice (164). Nevertheless, a cross-over study of intraduodenal infusion of sucralose (960 mg) in healthy individuals showed no difference in intestinal glucose absorption compared to saline infusion in combination with glucose (53). Additionally, intraduodenal infusion of sucralose (80 and 800 mg) was not found to stimulate GIP release compared to saline infusion in combination with glucose in healthy individuals (42). Notably, however, the measurement of intestinal glucose absorption in the latter study is less sensitive compared to the methodology applied in the rodent studies, as intestinal glucose absorption rate is indirectly measured by adding a non-metabolizable glucose analog to the intestinal perfusate (106). To date, no significant effects of artificial sweeteners on intestinal glucose absorption have been reported in humans.

### Insulin Secretion

The intake of nutrients is associated with a large set of sensory cues that enables the human body to prepare for metabolic digestion and utilization. Exposure to sweet-tasting sugars, even before ingestion, triggers physiological responses related to the release of insulin or incretin in order to reduce blood glucose levels. However, artificial sweeteners are not able to prepare the GI tract for digestion and utilization of nutrients as well as sugars (107, 165). Smeets and colleagues (107) have shown in a randomized crossover study in healthy individuals that there was no cephalic insulin response upon tasting of aspartame, while an early rise in insulin concentration was found when tasting glucose. Likewise, no cephalic response upon sucralose has been reported in a randomized crossover study in healthy individuals (52). Furthermore, while natural sugars are able to stimulate the secretion of incretins, thereby stimulating β-cells to secrete insulin, artificial sweeteners do not directly induce incretin secretion as this appears nutrient-dependent (39, 109, 110, 166). Moreover, insulin secretion is stimulated upon the interaction of both natural sugars and artificial sweeteners with sweet-taste receptors in pancreatic β-cells by initiating a signal transduction pathway via Ca^2+^ and cAMP-dependent mechanism (167). Taken together, this may suggest that artificial sweeteners stimulate insulin secretion less compared to natural sugars.

In agreement with this, the majority of acute and short-term (7–12 days) RCTs showed no significant effect of sucralose consumption or intravenous infusion on circulating insulin levels compared to water, glucose, sucrose, placebo (calcium carbonate), or saline infusion as control in healthy individuals (36, 37, 42, 51, 52, 168). Only three studies reported opposite findings, of which two studies found increased insulin levels after acute (48 mg) or long-term (4 weeks, 200 mg/day) sucralose consumption compared to water or placebo (unspecified) in obese or healthy individuals (169–171). The reasons for these discrepant findings are not clear but may be related to differences in study population or duration of the intervention. Moreover, Sylvetsky et al. (171) showed increased insulin levels after acute intake of a diet-beverage including sucralose, acesulfame-K, and aspartame compared to carbonated water (seltzer) in healthy individuals. Nevertheless, no differences in insulin levels were found upon water with sucralose consumption compared to water consumption alone, thereby indicating that the taste associated with diet soda or other ingredients may affect the insulin secretion. Furthermore, acute and longer-term (12–16 weeks) studies showed no effect of saccharin, acesulfame-K, steviol glycoside, and aspartame consumption on insulin levels in healthy, diabetic, overweight, or obese individuals (36, 39, 40, 43–45, 48, 172–174). Taken together, the available human data suggests that artificial sweeteners do not significantly affect insulin levels.

### Insulin Resistance

Insulin resistance is a major factor in the pathophysiology of T2DM, of which the pathogenesis involves the accumulation of ectopic fat and the activation of innate immune pathways, thereby interfering with insulin signaling and action (175). The artificial sweetener-induced gut microbiota dysbiosis has been linked to metabolic endotoxemia and the development of an inflammatory state, at least in rodents (89, 127, 176). Suez et al. (89) showed an altered host metabolism by downstream effects of microbiota in mice upon saccharin intake. The authors found enriched microbial pathways, associated with metabolic syndrome, in mice, including lipopolysaccharide (LPS) synthesis, which is a breakdown product of the outer membrane of Gram-negative bacteria (89, 177). Microbiota dysbiosis is considered to be related to the loss of gut mucosal integrity as the expression of tight junction proteins is reduced, among other mechanisms (176, 178). Therefore, LPS may translocate from the gut into the portal or systemic circulation, thereby able to stimulate the activation of pro-inflammatory macrophages and the secretion of pro-inflammatory cytokines (127, 177, 179–181). Other studies showed disrupted intestinal epithelial barrier *in vitro* using Caco-2 cells upon saccharin stimulation, whereas aspartame, acesulfame-K, and sucralose did not alter intestinal permeability (176). Similarly to the study of Suez et al. (89), other rodent studies showed increased LPS concentration, and subsequently enhanced inflammation, in mice upon saccharin consumption by interfering with the gut microbiota (127, 176). Regarding other artificial sweeteners, the intake of acesulfame-K (exceeding the ADI-dosage for humans by more than twice) or sucralose was found to enhance inflammation in mice, whereas steviol glycoside was found to reduce inflammation by attenuating LPS-induced pro-inflammatory cytokine production in Caco-2 cells and by regulating TLR2 and cytokine expression in *S. aureus*-infected mouse mammary gland (86, 182–184). This indicates that steviol glycoside possess anti-inflammatory properties, whereas saccharin, acesulfame-K, and sucralose may increase inflammation in rodent studies and *in vitro*. The resulting endotoxins and inflammatory cytokines are able to infiltrate peripheral tissues and release TNFα, IL-1β, and IL-6, which may interfere with insulin signaling and insulin-stimulated glucose uptake (185–187). Furthermore, inflammatory molecules may inhibit adipogenesis by constraining the hyperplastic expandability of adipose tissue (188). As a result, adipocyte turnover and adipose tissue expansion is reduced, leading to lipid overflow and fat accumulation in non-adipose tissues. This ectopic fat, as well as the accumulation of bioactive lipid metabolites, may disturb cellular function, ultimately contributing to insulin resistance and a reduced β-cell function, as described more extensively elsewhere (189).

Besides an enrichment of LPS synthesis, Suez et al. (89) showed an increase in SCFA production, through alterations in gut microbiota composition, in mice upon saccharin consumption. The authors suggested that the enhanced SCFA may serve as an energy source for the host or signaling molecules or substrates for gluconeogenesis, *de novo* lipogenesis and cholesterol synthesis (89). Counterintuitively, SCFA have most often been associated with positive health effects (140). SCFA were found to counteract LPS-induced inflammation by reducing pro-inflammatory cytokines and enhancing anti-inflammatory cytokines in murine macrophages (190). Furthermore, *in vitro* studies have found an attenuation of lipolysis upon SCFA stimulation in *3T3-L1* adipocytes, thereby reducing plasma free fatty acids (191–194). Likewise, rodent studies have demonstrated that SCFA may reduce intracellular lipid accumulation, thereby alleviating oxidative stress (195–197). In addition, as mentioned before, SCFA may affect energy metabolism, for instance via the enhancement of lipid oxidation in human studies (143). Repeatedly, artificial sweeteners have been found to increase lipid oxidation compared to sucrose in acute and long-term (10 weeks) RCTs in normal and/or overweight individuals (157, 158). Chern et al. (158) suggested that the difference in metabolism between sucralose and sucrose is attributed to the distinct carbohydrate content and the fact that sucrose is able to initiate carbohydrate-specific physiological responses, including the secretion of insulin and GLP-1. Taken together, it can be speculated that artificial sweeteners, to some extent, play a protective role in adiposity and insulin resistance by counteracting the LPS-induced inflammation and subsequent impairment of insulin signaling. However, it remains to be investigated whether the findings of Suez et al. (89) in mice are translatable to humans regarding the metabolic consequences of artificial sweetener-induced microbiota alterations. Furthermore, human evidence of the effects of artificial sweeteners on inflammation is currently lacking.

## Conclusion and Perspectives

The scope of this review was to review the physiological effects of artificial sweeteners on body weight control and glucose homeostasis, and to identify the controversies of the existing evidence between different artificial sweeteners surrounding their use. Although artificial sweeteners maintain the same palatability as natural sugars, the metabolic routes are different. Therefore, artificial sweeteners affect body weight and glucose homeostasis differently compared to natural sugars via underlying physiological processes comprising the gut microbiota, reward-system, adipogenesis, insulin secretory capacity, intestinal glucose absorption, and insulin resistance. The gut microbiota, in particular, may play a major role in the physiological effects of artificial sweeteners on body weight regulation and glucose homeostasis. There is mechanistic evidence that artificial sweeteners may induce gut microbiota dysbiosis, by altering the gut microbiota composition and function. Although different physiological processes are involved in the effect of artificial sweeteners on metabolic health, meta-analyses of RCTs or RCTs and prospective cohort studies suggest that artificial sweeteners may have a neutral effect on body weight and glycemic control, respectively, or may have a beneficial effect on long-term body weight regulation. Even though the majority of human studies report no significant effects of artificial sweeteners on body weight and glycemic control, it should be emphasized that the study duration of most studies was limited. Furthermore, unlike rodent studies, long-term studies investigating the underlying physiological effects body weight control on metabolic health of artificial sweeteners in humans are scarce and therefore warranted. Currently, within the European H2020 project SWEET (www.sweetproject.eu), a human multicenter study is ongoing which aims to investigate the use of artificial sweeteners within the context of a healthy lifestyle on body weight maintenance after weight loss and on metabolic health risk. Notably, artificial sweeteners are metabolized differently and may not all elicit the same metabolic effect as, for instance, components may affect the gut microbiota composition directly and others are easily digested and absorbed. Not all studies investigating the effects of artificial sweeteners on body weight control and glucose homeostasis take into account the different metabolic pathways of distinct artificial sweeteners. Therefore, human data on the effects of distinct artificial sweeteners are limited or lacking. The difference in metabolic fate of artificial sweeteners may underlie conflicting findings that have been reported related to their effects on body weight control, glucose homeostasis, and underlying biological mechanisms. Therefore, extrapolation of the metabolic effects of a single artificial sweetener to all artificial sweeteners is not appropriate.

In this regard, future studies should consider the metabolic pathways of different artificial sweeteners. Further (long-term) human research investigating the underlying physiological pathways of different artificial sweeteners on microbiota alterations and its related metabolic pathway is warranted to evaluate the potential impact of their use on body weight control and glucose homeostasis. Ultimately, it would be interesting to elucidate the impact of initial microbiota composition as a predictor for the response to artificial sweeteners in humans.

## Author Contributions

MP drafted and edited the manuscript. GG and EB conceptualized and reviewed the manuscript. All authors approved the final version of the manuscript to be published.

## Conflict of Interest

The authors declare that the research was conducted in the absence of any commercial or financial relationships that could be construed as a potential conflict of interest.
